# Comparative study of cell alterations in oral lichen planus and epidermoid carcinoma of the mouth mucosa

**DOI:** 10.1016/S1808-8694(15)30785-0

**Published:** 2015-10-19

**Authors:** Fernando Augusto Cervantes Garcia de Sousa, Thaís Cachuté Paradella, Adriana Aigotti Haberbeck Brandão, Luiz Eduardo Blumer Rosa

**Affiliations:** 1 Master's degree in oral biopathology (dental surgeon); 2 Master's degree in restorative dentistry (dental surgeon); 3 Assistant professor of general pathology, FOSJC/UNESP (Physician); 4 Adjunct professor of oral pathology, FOSJC/UNESP (dental surgeon)

**Keywords:** epidermoid carcinoma, lichen planus, mouth mucosa

## Abstract

Currently, much is discussed regarding the pre-malignant nature of mouth mucosa lichen planus.

**Aim:**

The present study aims at analyzing the alterations found in the epithelial cells present in the oral cavity lichen planus, comparing them to those found in epidermoid carcinoma.

**Materials and Methods:**

Histological cross-sections of oral lichen planus and epidermoid carcinoma, dyed by hematoxylineosin, were analyzed through light microscopy.

**Result:**

the most frequently found alterations in oral lichen planus were: an increase in the nucleus/cytoplasm relation (93.33%), nucleus membrane thickness (86.67%) and bi-nucleus or multinucleous (86.67%). The Student t test (alpha=5%) revealed a statistically significant difference between the average number of cell alterations in oral lichen planus (5.87±1.57) and in epidermoid carcinoma (7.60±1.81). As to the types of alterations, the chi-squared test also revealed statistically significant differences among the lesions assessed in relation to the following cell alterations: nuclear excess chromatism, atypical mitoses, cellular pleomorphism and abnormal cell differentiation (p<0.05).

**Conclusion:**

Despite the fact that in some cases, some pathologists may make mistakes in the histopathological diagnosis of oral lichen planus, the results obtained in this study show that the alterations present in oral lichen planus differ considerably from those seen in epidermoid carcinoma, thus showing how distinct these two diseases are.

## INTRODUCTION

Since 1910, when the first case of gingival cancer was reported in a patient with oral lichen planus (OLP), the latter has become the focus of much controversy. Many studies have attempted to assess the malignant transformation potential of OLP. These studies have suggested that a lesion originally diagnosed as OLP has a 6.51% possibility of undergoing malignant transformation in time;^1^, ^2^, ^3^, ^4^, ^5^ based on these studies, the World Health Organization (WHO) has classified OLP as a potentially malignant disease.^6^

Some authors, however, argue that such transformation has not been sufficiently documented to justify this classification. According to these authors, more precise criteria are needed to diagnose OLP precisely, especially from a histopathological standpoint. Most of the cases of malignant transformation would thus not be considered as such, since in these cases there probably were histopathological signs suggesting a malignancy at the moment of the initial diagnosis, which would void the hypothesis of OLP.^7^, ^8^

Van der Meij and Van der Waal^9^ (2003) illustrated this difficulty in the diagnosis of OLP. These authors found that there was no consensus in the histopathological diagnosis of 42% of cases in which the clinical diagnosis was clear. This is probably because the inflammation present in OLP may cause cell alterations similar to those seen in epithelial dysplasia or in epidermoid carcinoma.^10^

In this context, the purpose of this study was to analyze the changes in epithelial cells of OLP, and to compare such changes with those found in the epidermoid carcinoma; the intention was to seek for similarities and differences to facilitate the histopathological diagnosis and establish its inflammatory nature.

## MATERIAL AND METHOD

After reviewing OLP and epidermoid carcinoma cases diagnosed at the Serviço de Patologia Cirurgica da Faculdade de Odontologia de Sao Jose dos Campos, UNESP, from 1995 to 2005, thirty cases of each lesion were randomly selected. Three independent examiners reassessed these cases to confirm the initial histopathological diagnosis. If any doubts remained, the case was immediately replaced.

Eisenberg's^7^ (2000) histopathological criteria were used as essential for the diagnosis of OLP, as shown on Table 1. Additionally, only those cases which generated no doubts about the diagnosis of OLP, in patients that did not smoke or take alcoholic beverages, were included in this study.

**Table 1:** Histological criteria for the diagnosis of OLP^7^

Essential findings
• liquefied baseline layer
• intense lymphocyte infiltrate in layers underlying the epithelium, with effacement of the baseline layer
• normal epithelial cell maturation
**Other findings (non-essential)**
• interpapillary crests in a “sawtooth” shape
• hyperparakeratosis
• Civatte bodies
• separation of the epithelium of the lamina propria
**Exclusion criteria**
• cells with large and/or hyperchromatic nuclei
• presence of dyskeratosis
• increased number of mitoses or atypical mitoses
• projection of epithelial “drop-like” cones
• absence of liquefied baseline layer
• loss of epithelial stratification
• heterogeneous inflammatory infiltrate
• extension of infiltrate to deeper layers
• perivascular infiltrate

Paraffin blocks with the biopsied samples were separated for each case. New 5μm slices were made of these samples and placed on clean glass slides for hematoxilin-eosin (HE) staining.

Two independent examiners analyzed the histological sections under light microscopy. Cell alterations were investigated in each of the cases, according to the following criteria:

a) increased nucleus/cytoplasm ratio;
b) hyperchromatic nuclei;
c) irregular distribution of chromatin;
d) thickening of the nuclear membrane;
e) loss of cell adhesion;
f) increased size and number of nucleoli;
g) bi- or multinucleation;
h) atypical mitoses;
i) cell pleomorphism;
j) abnormal cell differentiation.

If examiners differed as to the presence or absence of any criterion, a third examiner evaluated the case in question under similar conditions; the majority opinion prevailed.

Student's t test and the chi-square test were used for the statistical analysis; the significance level was 5%.

Finally, all of the procedures described above were authorized by the Research Ethics Committee of the Faculdade de Odontologia de Sao Jose dos Campos - UNESP (protocol number 008/2006-PH/CEP of 14 March 2006).

## RESULTS

At the end of the analysis, a mean 5.87 (±1.57) cell alterations per case were found in OLP; this mean was 7.67 (±1.81) in epidermoid carcinoma cases. Student's t test showed that there was a statistically significant difference between these mean numbers of cell alterations (p<0.05).

The most frequent alterations in OLP were: increased nucleus/cytoplasm ratio (93.33%), thickening of the nuclear membrane (86.67%), and bi- or multinucleation (86.67%). Atypical mitoses or abnormal cell differentiation were not seen in any of the cases.

**Figure 1:**
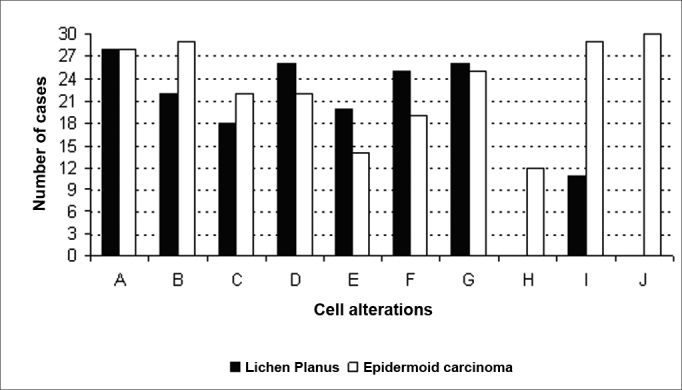
Frequency of cell alterations in OLP and the epidermoid carcinoma (A - increased nucleus/cytoplasm ratio; B - hyperchromatic nuclei; C - irregular distribution of chromatin; D - thickening of the nuclear membrane; E - loss of cell adhesion; F - increased size and

In epidermoid carcinoma cases, all of the alterations were present at an increased frequency; of these, abnormal cell differentiation (100%), hyperchromatic nuclei (96.67%), and cell pleomorphism (96.67%) were the most frequent (Figure 1).

The chi-square test revealed a statistically significant difference between OLP and the epidermoid carcinoma in the following cell alterations: hyperchromatic nuclei, atypical mitoses, cell pleomorphism, and abnormal cell differentiation (p<0.05).

## DISCUSSION

Although OLP is relatively common, its potential for malignant transformation has always been a controversial issue. Case surveys undertaken recently in various countries have stimulated this controversy even further by suggesting that the malignant transformation rate may range from 0.65% to 6.51%.^1^, ^2^, ^3^, ^4^, ^5^

Some authors, however, believe that malignant transformation has not been sufficiently documented to justify the classification of OLP as a precancerous lesion, as proposed by the WHO.^6^ For these authors, both clinical and histopathological criteria for diagnosing OLP with greater precision are lacking; this situation questions the cases of malignant transformation of OLP reported in the literature.^7^, ^8^

Our study revealed that cell alterations, such as an increased nucleus/cytoplasm ratio, irregular distribution of chromatin, thickening of the nuclear membrane, loss of cell adhesion, increased size and number of nucleoli, and bi- or multinucleation may be seen both in OLP and in the epidermoid carcinoma.

This is probably because some of the inflammatory features are similar to those seen in malignancies, particularly those that indicate increased proliferation due to the many growth factors released to epithelial cells in chronic inflammation.^10^ Mignogna et al.^11^ (2004) have suggested that such alterations should be analyzed with greater care, since there is current evidence showing that chronic inflammation may create a cytokine-based microenvironment capable of affecting cell lifetime, growth, proliferation and differentiation, which may lead to cancer promotion and progression.

Thus, it is not hard to understand that even experience pathologists may mistake epithelial dysplasia with OLP, especially in cases where there is more intense inflammation. This situation, unfortunately, is not rare; the WHO has recently defined another condition known as lichenoid dysplasia. This condition is similar to OLP, but shows varied degrees of epithelial dysplasia, and thus a higher possibility of malignant transformation.^7^, ^8^^–^^,^^10^^,^^12^, ^13^, ^14^, ^15^ Furthermore, other conditions may also present similar histopathological findings as those found in OLP, including lichenoid reactions, lupus erythematosus, leukoplasia, erythroleukoplasia, and proliferative verrucous leukoplasia.^10^

Our results, however, suggest that atypical mitoses and abnormal cell differentiation are inherent to the epidermoid carcinoma, and that hyperchromatic nuclei and cell pleomorphism are seen more often in this disease, rather than in OLP. Thus, such changes might help separate OLP from other dysplastic diseases, which are potentially precancerous.

If, on the one hand, our data support the hypothesis that many of the cases of malignant transformation described in the literature are due to an initial failure in diagnosis, on the other hand, our data also show that there are significant histopathological differences between OLP and the epidermoid carcinoma. The most frequent alterations found in OLP were an increased nucleus/cytoplasm ratio (93.33%), thickening of the nuclear membrane (86.67%), and bi or multinucleation (86.67%); the most frequent alterations found in the epidermoid carcinoma were abnormal cell differentiation (100%), hyperchromatic nuclei (96.67%), and cell pleomorphism (96.67%).

Finally, it should be noted that although recent studies have proposed using more advanced methods for studying the potential for malignant transformation of OLP,^16^, ^17^, ^18^, ^19^ more detailed knowledge of its histopathological features, and especially of its clinical progression, remain essential to understand the true nature of this disease.

## CONCLUSION

Although in some cases pathologists may have difficulties in the histopathological diagnosis of OLP, our results show that the alterations in OLP differ considerably from those found in the epidermoid carcinoma, revealing the fact that these conditions are markedly different.
